# Analysis of the usage of continence pads and help-seeking behavior of women with stress urinary incontinence in Poland

**DOI:** 10.1186/s12905-015-0238-6

**Published:** 2015-09-30

**Authors:** Magdalena Emilia Grzybowska, Dariusz Wydra, Jerzy Smutek

**Affiliations:** Department of Gynecology, Gynecologic Oncology and Gynecologic Endocrinology, Medical University of Gdańsk, Kliniczna 1a, 80-402 Gdańsk, Poland; Department of Perinatology, Medical University of Gdańsk, Kliniczna 1a, 80-402 Gdańsk, Poland

**Keywords:** Stress urinary incontinence, Urinary leakage, Incontinence pads, Female, Disease progression, Health care seeking behavior, Age at onset, Urodynamics

## Abstract

**Background:**

Urinary incontinence (UI) in women is a chronic disorder which has a negative impact on health-related quality of life. Only 45 % of the affected individuals report the problem with continence to their doctor. The aim of the study was to assess the duration of stress urinary incontinence (SUI), time from disease onset to the first medical consultation and in-depth diagnosis, as well as the need for using continence pads in various grades of SUI.

**Methods:**

We conducted a cross-sectional study in women who presented at the urogynecologic ambulatory center and reported urinary incontinence. A total of 420 subjects were interviewed and underwent urogynecologic and urodynamic examinations. A group of 147 patients with urodynamic SUI was enrolled in the study.

**Results:**

All women were graded according to the Stamey severity score: grade 1 – 56 (38.1 %), grade 2 – 68 (46.3 %), and grade 3 – 23 (15.6 %). Mean time elapsed between disease onset and presentation at the urogynecologic ambulatory center was 17.4 ± 11.8 years (grades 1, 2 and 3 for 11.6 ± 11.8, 14.9 ± 10.8, and 22.2 ± 12.1 years, respectively; *p* = 0.0002). Patients with SUI started perceiving their condition as a problem 4.7 ± 5.4 years before referral to urodynamics; 58.3 % of the SUI patients reported their problems with continence to a physician. Average time between the onset of UI symptoms and seeking medical help was 13.28 ± 12.3 years. Mean duration of using continence pads during the day was 4.2, 5.4 and 10.2 years in grades 1, 2 and 3, respectively (*p* = 0.0002). The number of patients using continence pads in and outside the home, as compared to outside only, was: grade 1 – 44.6 % vs. 28.6 %, grade 2 – 77.6 % vs. 13.4 %, and grade 3 – 86.4 % vs. 9.1 % (*p* = 0.004). Mean use of continence pads at night was 3.3, 6.1, and 9.1 years in grades 1, 2 and 3, respectively. The differences were not statistically significant. Protective continence products were used at night by 26.7 % of the SUI patients: 16.1 %, 25 % and 59.1 % in grades 1, 2 and 3, respectively (*p* = 0.004).

**Conclusions:**

Women with SUI delay seeking medical help for over a decade. The severity of SUI is associated with duration and increased use of continence pads.

## Background

Urinary incontinence (UI) is a chronic condition affecting approximately 16.8 % of the Polish female population but, due to the embarrassing nature of the problem, the data are believed to be underestimated [1]. According to other epidemiological and clinical studies, 10-44 % of all women develop UI or other urogynecologic problems, which is associated with several risk factors or contributing variables [2–4]. These data suggest that UI should be perceived as a health problem which markedly influences the lives of the affected people.

Depending on patient cultural background, UI has been treated either as a symptom of aging (49.5 %), or an inevitable consequence of childbirth (51.9 %) [5]. According to Kinchen et al. [6], only 38 % of women mention their current problems with UI during a doctor’s appointment, whereas the conversation about UI is initiated by the physician in only 7 % of the cases. Thus, over half of American women will remain silent about their struggle with incontinence during a medical appointment. Some women are convinced they can manage with the problem themselves (22.2 %), others do not believe in treatment possibilities (13.7 %) [7]. The greater the severity and frequency of UI symptoms, the more willingly the patients talk about UI. According to Hannenstad et al. [8], only every fourth UI patient seeks help.

Incontinent women turn to doctors of various specialties. According to data from the U.S., they usually consult their GP (42.9 %), an obstetrician/gynecologist (35.1 %), and an internist (10.9 %), whereas only 4.4 % would address a urologist [9]. Mean age of women with UI who seek medical care is significantly higher than their peers who do not seek help (56.9 vs. 51.8, *p* < 0.01) [6, 8, 10]. Patients with urgent or mixed urinary incontinence are more likely to seek help than women with stress urinary incontinence (SUI) [9, 10]. Women who get regular checkups or those with comorbid diseases seek treatment more often [6]. Undoubtedly, the feelings of shame or guilt are the reason for lack of doctor-patient communication. According to Saleh et al. [5], it applies to 54 % of women with UI. A similar proportion of female patients consider UI to be too shameful to discuss it with their husbands (56 %). Numerous studies confirmed that patients seeking medical help have significantly lower self-perceived health-related quality of life (HRQoL) in comparison to women who do not report the problem to their doctor [10, 11].

According to Temml et al. [12], 65.7 % of the affected women report a negative impact of UI on HRQoL. Changes in the behavior relate to most areas of everyday activity: social, psychological, occupational, domestic, physical, and sexual [13]. HRQoL impairment significantly correlates with the frequency of urinary leakage episodes, severity of incontinence, and the need to use continence pads (*p* < 0.01) [12]. UI in the elderly causes social isolation, increases psychological distress and costs (continence products, detergents), and often contributes to a growing number of admissions to nursing homes [6]. The use of incontinence products generates great additional costs and constitutes an additional financial burden both, for the patients and the health care system. In the U.S., approximately 70 % of the total cost of UI (of $12.4 billion) is related to routine care, e.g. absorbent products [14]. In Poland, the ratio are different, the annual UI-related cost amounts to 2 billion PLN, including 270 million PLN (13.5 %) for incontinence products alone.

The primary aim of our study was to measure time elapsed before UI women decide to consult a doctor and receive in-depth diagnosis. The secondary aim was to analyze the usage of continence pads in various grades of SUI. It is an important issue and it may help to calculate the disease-related costs before effective treatment is applied.

## Methods

We conducted a cross-sectional study in women who presented at the urogynecologic ambulatory department between 2001 and 2006 and reported problems with UI. A detailed medical history was taken with the emphasis on UI (including a thorough analysis of the current frequency of urine leakage, situations in which they occur, current need for using continence pads, and the micturition pattern). The patients also submitted information about the time of UI onset, symptom severity, and treatment received so far. Each subject was asked to prepare a 48-h voiding diary. Urogynecologic examination was conducted and pelvic organ prolapse was assessed according to the Pelvic Organ Prolapse Quantification (POP-Q) system. Negative urine culture was the requirement for urodynamic examination, performed with the Solar® equipment (Medical Measurement System, The Netherlands) and in accordance with the standards of the International Continence Society [15]. The inclusion criterion was urodynamically confirmed pure SUI, patients with mixed and urge UI were excluded. In a group of 420 patients with UI, 264 women were diagnosed with SUI. As the study was a part of the research on HRQoL in patients with UI, participants who refused to consent were excluded. Thus, a group of 147 women were assessed (response rate of 55.7 %). After the diagnosis, the patients were graded according to the Stamey score, i.e. grade 1 - urine leakage on strong physical effort (coughing, sneezing), grade 2 - medium effort (change of position) and grade 3 – urine leakage on minimal effort (lying position) [16].

The study was approved by the Ethics Committee of the Medical University of Gdańsk, and all patients gave their verbal informed consent to take part in the study. The privacy of participants was maintained. The Declaration of Helsinki was followed.

### Statistical analysis

The statistical analysis was performed with STATISTICA version 10. All continuous variables were expressed as mean ± SD. Categorical variables were expressed as percentages of the total group. A *p*-value of <0.05 was considered as statistically significant. Differences in continuous variables were initially assessed with Levene’s test for homogeneity of variance. Analysis of variance was performed for more than two groups, the Kruskal-Wallis test was used for variance inequality or lack of variance homogeneity. Post-hoc tests were employed for statistically significant results. The comparison of the ordinal variables of the two groups was performed with the Mann–Whitney *U* test, and in case of more than two groups with the Kruskal-Wallis test and post-hoc analysis, if appropriate. Comparisons of categorical variables were performed using Chi-square test, Chi-square test with Yates continuity correction or Fisher’s exact test.

Based on data from the literature [6] it may be expected, that frequency of reporting the problem of UI to the doctor will be about 38 %. To achieve required precision of 8 % required sample size was 142 patients.

## Results

Table 1 presents the percentage of patients with particular grades of SUI according to the Stamey score. As far as POP (evaluated with the POP-Q system) is concerned, stage 0 was diagnosed in 9 (6.1 %), stage I in 24 (16.3 %), stage II in 103 (70.1 %), and stage III in 11 (7.5 %) patients. No stage IV POP-Q cases were found. Mean age was 53.89 ± 9.5 years (range 24 – 83), mean BMI was 27.0 kg/m^2^ ± 4.8. In the analyzed group, 99.3 % of the women were parous, 3 reported no vaginal delivery, among them 2 were nulliparous. Fifty patients (34 %) were premenopausal and 97 (66 %) were postmenopausal. Mean age at menopause was 49.16 ± 4.8 years (range 30–58). Thirty-four of the postmenopausal patients reported past or current use of hormone replacement therapy.

**Table 1:** Pre- and postmenopausal patients (Stamey incontinence score of SUI)

Grades of SUI		Premenopausal patients		Postmenopausal patients	
	n	n	%	n	%
1	56	23	46.0	33	34.0
2	68	19	38.0	49	50.5
3	23	8	16.0	15	15.5
Total	147	50		97	
p		>0.05			

p - level of significance for Chi-square test with Yates continuity correction

In the postmenopausal group, 47 patients reported symptom onset after menopause: 15 - within a year of the last menstrual bleeding, 13 - in the following year, 5 - within 5 years, and 14 - in the subsequent years. UI occurred after menopause within 7.5 ± 7.9 years (range 0–29 years).

Study subjects have suffered from UI for 17.4 ± 11.8 years (range 1–55 years). The duration of UI, in particular SUI grades 1–3 was 11.6 ± 11.8, 14.9 ± 10.8, and 22.2 ± 12.1 years, respectively. Significantly longer duration was found in patients with grade 3 as compared to grades 1 or 2 (*p* = 0.002 and *p* = 0.00003, respectively).

Ten patients (6.8 %) suffered from symptoms of stable intensity. As a result of the urodynamic studies, 8 of them were diagnosed with grade 1 and 2 with grade 2 of SUI. The remaining 137 patients (93.2 %) reported progression of symptoms in the course of 5.27 ± 5.3 years (range 0.5–26 years) before presenting at an urogynecologic ambulatory center. The disease had been a problem for 4.7 ± 5.4 years before the affected women underwent urodynamic examination (range 0.5–30 years).

Sixty (41.6 %) SUI patients presented directly at an urogynecology center, while 84 (58.3 %) reported problems with UI to their gynecologist, urologist or GP (3 patients did not submit their data). Average time between symptom onset and reporting UI to a physician was 13.28 ± 12.3 years, and between informing the doctor and the diagnostic tests was 3.87 ± 4.6 years (range 0.5–22 years). Time elapsed between consulting the physician and thorough diagnostic tests also did not differ significantly in particular grades of SUI (Table 2).

**Table 2:** Time from onset to first medical consultation of UI in SUI patients

Grades of SUI	Patients who revealed the problem to their doctor		Time between disease onset and informing the doctor	Time between informing the doctor and thorough diagnostic tests
	(n = 84)		(years ± SD)	(years ± SD)
	n	%		
1	33	60.0 %	13.1 ± 12.5	3.3 ± 4.6
2	36	52.9 %	12.8 ± 11.5	4.5 ± 4.9
3	15	71.4 %	23.0 ± 12.8	3.5 ± 3.4
p	>0.05^a^		0.002^b,c^	0.19^b^

^a^level of significance for Chi-square test

^b^level of significance for Kruskal-Wallis test

^c^Duncan post-hoc analysis in particular grades of SUI: 1 *vs*. 3, *p* = 0.0002, 2 *vs.* 3, *p* = 0.003

In our study, time elapsed between symptom onset and informing the doctor depended significantly on the severity of incontinence at diagnosis. Grade 3 SUI patients delayed reporting their problem to the doctor significantly longer than women with grades 1 and 2 SUI (*p* = 0.0002 and 0.003, respectively).

Patients with higher grades of SUI had to use continence pads during the day significantly longer (grade 1 > 4, grade 2 > 5, and grade 3 > 10 years). Women using continence pads in and outside the home had a significantly higher severity of incontinence than those using protective pads only outside the home (*p* = 0.004). Grade 1 was most frequently observed in subjects using continence pads only outside the home, whereas 4.8 % of SUI patients, all grade 1, needed no incontinence protection (Table 3).

**Table 3:** Use of continence pads during the day in SUI patients

Grades of SUI		Methods of protection against UI						Duration of use of continence pads
		continence pads				change of underwear	none	
		in and outside the home	only outside the home	occasionally	hygiene reasons			
	n	n (%)	n (%)	n (%)	n (%)	n (%)	n (%)	(years ± SD)
1	55	25 (44.6)	16 (28.6)	1 (1.8)	3 (5.4)	4 (7.1)	7 (12.5)	4.2 ± 6.5
2	68	52 (77.6)	9 (13.4)	2 (3.0)	-	4 (6.0)	-	5.4 ± 3.9
3	22	19 (86.4)	2 (9.1)	1 (4.5)	-	-	-	10.2 ± 8.9
Total	145	96	27	4	3	8	7	
p		0.004^a^						0.0002^b^

2 patients did not provide information

^a^level of significance for the Mann–Whitney *U* test

^b^level of significance for ANOVA rank Kruskal-Wallis test, independent variable severity of SUI

The use of continence pads at night (in years) increased with the severity of SUI, but the difference was not statistically significant. Thirty-nine patients (26.7 %) reported the need for sanitary protection at night, including 6 patients using adult diapers. Protective pads at night were mostly used by grade 3 SUI women. Among subjects reporting the use of continence pads at night only 23.1 % were grade 1, whereas 43.6 % and 33.3 % were grades 2 and 3, respectively (Table 4).

**Table 4:** Use of continence pads at night in SUI patients

Grades of SUI		The methods of protection against UI					Duration of use of continence pads
		protection at night		hygiene reasons	occasionally	change of underwear	
		yes	no				
	n	n (%)	n (%)	n (%)	n (%)	n (%)	(years ± SD)
1	56	9 (16.1)	35 (62.5)	1 (1.8)	10 (17.8)	1 (1.8)	3.3 ± 2.5
2	68	17 (25.0)	41 (60.3)	-	9 (13.2)	1 (1.5)	6.1 ± 8.5
3	22	13 (59.1)	7 (31.8)	-	2 (9.1)		9.1 ± 7.6
Total	146	39	83	1	21	2	
p		0.004^a^					0.09^b^

1 patient did not provide information

^a^level of significance for the Mann–Whitney *U* test

^b^level of significance for ANOVA rank Kruskal-Wallis test, independent variable severity of SUI

## Discussion

In our study, over half (58.3 %) of the SUI patients reported continence problems to their doctor, while the rest received information about diagnostic tests and therapy from media or friends. In the U.S., that number is estimated at 38–45 % [6, 17]. According to Kinchen et al. [6], patients with severe UI are three times more likely to seek medical help than their peers with moderate severity of UI (OR 4.13, 95 % CI 1.35–12.59 vs*.* OR 1.39, CI 1.00–1.94). In our study, there were no significant differences in the rate of women seeking medical advice in grades 1 and 2 SUI, but we observed a tendency to inform the doctor about continence problems much more frequently in grade 3 patients. Kinchen et al. [6], confirmed that an increase in the number of women reporting the disease to the doctor corresponds to disease duration. Our study showed that women with grade 3 SUI delayed contact with the doctor the longest, as compared to their peers with grades 1 and 2 SUI.

The prevalence rate of UI in postmenopausal women ranges from 34.6 to 50 % [1, 18]. In our study, postmenopausal patients accounted for 66 % of the SUI group. Premenopausal patients developed grade 1 SUI more often than their postmenopausal peers (46 and 34 %, respectively), grade 2 was more common among postmenopausal women (50.5 vs. 38 %), but the difference was not statistically significant. A similar percentage of patients in both groups were diagnosed with grade 3 SUI.

Our research was conducted in the first decade of this century, i.e. at the time of the awareness campaign about urogynecology in Poland and growing access to urodynamic testing, resulting in new diagnostic possibilities. Women who had been suffering from UI for years decided to seek help, probably due to escalating symptoms connected with disease progression. UI is accompanied by fear of public manifestation of symptoms and potentially shameful situations, leading to deteriorated quality of life [10]. Patients frequently admit that previously used methods (e.g. fluid restriction, frequent voiding, keeping the pelvic floor muscles in constant tension, etc.) are no longer sufficient to fully control the symptoms of UI, which prompts them to seek help.

U.S. studies confirmed the use of continence pads in 42 %, frequent emptying of the bladder in 33.5 %, and fluid restriction in 23.3 % of the affected individuals [9]. In our study, among patients presenting for urogynecologic examination, the rates of women constantly using continence pads during the day and at night were 66.2 and 26.7 %, respectively. We performed a detailed analysis of the circumstances and types of continence pads used by SUI women. None of the patients with SUI grade 2 and higher risked using no protection during the day. The group of patients who changed underwear after leakage were usually people of lower income. Women with excellent preventive methods, who understand the pathophysiology of UI and can predict situations with leakage, were occasional users of incontinence products. Diokno et al. [9], divided coping strategies for UI into defensive or intended to hide urinary incontinence. Methods aiming at preventing urine leakage cause limitation of physical and social activity. Urine leakage can be hidden by frequent washing, change of underwear, use of absorbent materials (protective perennial pads, diapers), or wearing dark and long clothes to hide the stains [5, 9].

SUI has been suggested to be progressive, but the literature lacks conclusive reports. Lifford et al. [19], investigated females with UI (aged 54–79) for 2 years and proved disease progression in 32.1 %, reduction of symptoms in 8.9 %, and complete symptom remission in 2 % of the women. However, their analysis did not involve pelvic organ prolapse assessment, which may mask or reduce the severity of SUI, leading to occult UI. Our analysis revealed a significant relationship between disease duration and severity. Mean disease duration in grades 1–3 was 11.6, 14.9, and 22.2 years, respectively.

Irwin et al. [20], who conducted a meta-analysis of articles published between 1990–2009, support the hypothesis of dynamic development and persistence of UI symptoms. They draw attention to the lack of uniformity in collecting information from the affected patients, the form (questionnaire) of the population studies, and assessing the discomfort by means of a self-report. Herzog et al. [21], during a 2-year follow-up of patients >60 years of age, confirmed the annual incidence of UI in 20 % of the study population. Additionally, Townsend et al. [17], examined a group of women between 36 and 55 years and recognized UI as a dynamic process, estimating that 1 out of 7 continent women will have suffered from UI, at least once a month, by the end of a 1-year observation period.

An advantage of our study is the complete diagnostic process involving urodynamic tests, which also allowed us to analyze and compare the symptoms reported by patients. Disease duration is directly related to how long (in years) continence materials have been used, which in our study was 4.2, 5.4 and 10.2 during the day in grades 1–3 of SUI, respectively. Women using incontinence protection only outside the home are much more likely to be grade 1 SUI according to the Stamey score, whereas patients using continence pads at night are significantly more frequently diagnosed with grade 3 SUI. In the questionnaire survey by Diokno et al. [9], involving a large population of American women with various types of UI, 84 % of the subjects reported the use of continence materials, including 5.2 % using adult diapers. In our study group the results were similar, with only 6.9 % of women reporting no incontinence protection during the day but 4.1 % using diapers at night. The everyday usage of continence pads is not only a significant cost, but also a factor strongly contributing to deteriorated HRQoL in UI patients [12].

Cross-sectional design and patient recall for data collection about the beginning of UI constituted two major limitations of our study. However, women very often associate the onset of UI with particular points in their lives, i.e. pregnancy, childbirth, menopause, which facilitates time estimation.

According to Kinchen et al. [6], less than half of UI patients admit to incontinence problems during check-up. Some authors draw attention to the fact that not all doctors have the necessary knowledge about the issue, and sometimes they avoid the topic for fear of making the patients uncomfortable. In a study conducted among doctors in Oklahoma, 50 % of GPs and 73 % of internists regarded their knowledge about UI as insufficient [22]. In light of the above mentioned facts, it seems possible that the actual incidence rates of UI differ from the estimates reported by epidemiological studies.

A new treatment option by Sjostrom et al. [23], without face-to-face contact with a physician, could be introduced to avoid UI-related embarrassment. An Internet-based treatment would exclude the necessity to talk to a doctor and offer an alternative to the affected individuals.

## Conclusions

Our data show that women with SUI delay consultation and proper treatment of their disability for over a decade. It can be connected with disease specificity and lack of social acceptance for this condition. It may also be a clue for health providers to implement programs which will inform the society about these problems, similarly to obesity, diabetes, or other diseases. Our cross-sectional data indicate that SUI is more severe in women with longer SUI duration. Long-term prospective studies are needed to understand changes in SUI severity over time. Our study also demonstrated that not enough attention is paid to QoL before patients seek medical advice on UI for the first time. SUI severity is associated with disease duration and increased use of absorbent pads. Thus, the question about the need for continence pads can be an important element of a screening interview. The increasing emphasis on QoL assessment ought to make physicians ask their patients about lower urinary tract symptoms routinely.

## Abbreviations

BMI: Body mass index
HRQoL: Health-related quality of life
OR: Odds ratio
POP-Q: Pelvic Organ Prolapse Quantification
QoL: Quality of life
SUI: Stress urinary incontinence
UI: Urinary incontinence
